# Developing Functional Relationships between Soil Moisture Content and Corn Early-Season Physiology, Growth, and Development

**DOI:** 10.3390/plants12132471

**Published:** 2023-06-28

**Authors:** Ranadheer Reddy Vennam, Purushothaman Ramamoorthy, Sadikshya Poudel, Kambham Raja Reddy, William Brien Henry, Raju Bheemanahalli

**Affiliations:** 1 Department of Plant and Soil Sciences, Mississippi State University, Mississippi State, MS 39762, USA; rv370@msstate.edu (R.R.V.); sp2306@msstate.edu (S.P.); krreddy@pss.msstate.edu (K.R.R.); wbh3@msstate.edu (W.B.H.); 2 Geosystems Research Institute, Mississippi State University, Mississippi State, MS 39762, USA; purush@gri.msstate.edu

**Keywords:** biomass, early-season drought, physiology, root-to-shoot, soil moisture content, stress tolerance

## Abstract

Drought is a severe threat to agriculture production that affects all growth stages of plants, including corn (*Zea mays* L.). Any factor affecting early seedling growth and development will significantly impact yield. Despite the recurrence of low rainfall during the growing seasons, corn responses to different early-season soil moisture content levels have not been investigated. In this study, we investigated how corn morpho-physiological and biomass traits responded to varied soil moisture content during the early vegetative stage. Two corn hybrids were grown in a pot-culture facility under five different soil moisture treatments (0.15, 0.12, 0.09, 0.06, and 0.03 m^3^ m^−3^ volumetric water content, VWC) to assess the growth and developmental responses to varied soil moisture content during early-season growth (V2 to V7) stage. Sub-optimal soil moisture content limited plant growth and development by reducing physiological and phenotypic expression. Stomatal conductance and transpiration were decreased by an average of 65% and 59% across stress treatments relative to optimum conditions. On average, soil moisture deficit reduced the total leaf area by 71% and 72% compared to the control in ‘A6659VT2RIB’ and ‘P1316YHR’, respectively. Shoot and root dry weights were reduced by 74% and 43% under 0.03 m^3^ m^−3^ VWC. An increase in the root-to-shoot ratio was noticed under low VWC conditions compared to the control. Based on the stress tolerance index, the physiology and leaf growth parameters were more sensitive to soil moisture deficit. Our results highlight the impact of sub-optimal soil moisture on physiology and morphological traits during early-season growth. ‘P1316YHR’ demonstrated better physiological performance under stress conditions, while ‘A6659VT2RIB’ produced relatively better root growth. The findings suggest that biomass partitioning between shoot and root components is dynamic and depends on stress intensity. The current findings can help to prioritize traits associated with the early-season drought tolerance in corn. The functional relationships developed between soil moisture content and growth and developmental responses can be integrated into corn crop modeling to allow better irrigation management decisions.

## 1. Introduction

Corn is an important cereal crop grown in over 170 countries [1]. It significantly contributes to global cereal crop production, with an annual output of 1.2 billion metric tons from an estimated acreage of 202 million hectares [2,3]. The United States is the leading producer of corn, accounting for more than 30% of the global production with an acreage of 34 million hectares. However, only 5 million hectares of the total corn cultivation area is irrigated, indicating that nearly 85% of corn production in the United States is under rainfed conditions [4]. When crop production depends on rainfall, the increased frequency of drought episodes during the growing season due to climate change affects growth and yield. Using advanced breeding, agronomic, and management practices, a yield potential of up to 12 tons per hectare has been achieved for corn under favorable growing conditions [5,6]. However, climate change-induced weather fluctuations, such as drought and flooding, subject the crops to sub-optimal growing conditions during the season. On average, over 60% of corn-growing areas experience drought stress during one or more growth stages [7]. For instance, drought stress, after planting or at early vegetative growth, adversely impacts plant stand per unit area and early seedling vigor [8].

Approximately 36% of corn-growing regions encountered moderate to extreme drought conditions during the vegetative stage in the United States, which is a condition undesirable for better crop establishment [9]. Corn is more susceptible to drought stress than other cereal crops [10,11]. The degree to which corn growth and development are adversely affected depends on the drought intensity, duration, and growth stage [12]. Plants undergo various physiological and morphological changes to withstand drought stress at the early vegetative stage [13]. Early-season vigor is a desirable trait for stress tolerance [14,15] and is considered one of the traits significantly associated with yield potential under non-stress and stress conditions [16,17]. A rapid canopy growth or higher biomass at the vegetative stage has many advantages, such as (i) early vigor enables rapid ground cover, which prevents soil water evaporation or allows plants to utilize available topsoil moisture efficiently before it evaporates, (ii) profuse root proliferation supports the shoot system, (iii) vigorous canopy can suppress or compete with weeds, and (iv) higher canopy level promotes photosynthesis and nutrient uptake. Therefore, traits associated with early plant vigor are the desirable proxy for late-season plant health and productivity [16,18].

Plant water relations are profusely governed by available soil moisture content, directly influencing stomatal conductance and photosynthesis [19]. Modified physiological processes cause changes in leaf development, leaf area, and dry weights [20,21,22]. Reducing leaf area also negatively influences the photosynthetic potential and pigment composition by limiting the surface area for CO_2_ assimilation and light absorption [23]. The benefits of a robust root system have been reported in several crops, including corn [24,25]. Stresses modify resource allocation between shoots and roots, as shown with cold stress [26], high temperature [27], and salt [28].

On the other hand, plasticity in roots allow plants to adapt to changes in the growing environment. Among all root traits, higher root biomass is essential for seedling survival, and their vigor is closely associated with above-ground biomass and yield [29]. Despite the significant impact of drought stress on the shoot and root traits, root-to-shoot responses to different moisture stress levels have not been investigated in corn. Sub-optimal soil moisture (drought stress) at the early growth could strongly limit plant functions and biomass production. Besides the growth, the effects of drought stress on physiology and root responses in corn to different soil moisture levels remain relatively less explored and require extensive research. These modifications to promote survival under drought conditions adversely affect the phenotypic expression in plants [30]. Depending on the severity of the stress, trait response may vary with genetics. For example, if the long-term drought persists, plants spend energy on survival mechanisms by conserving water (low stomatal conductance). At the same time, maintaining growth and vigor is the primary focus under short-term drought conditions [31].

Hence, understanding corn responses to different moisture stress conditions during early vegetative growth provides insight into the interrelationship between plant physiological and morphological responses. Furthermore, identifying optimum and minimum moisture requirements during early vegetative growth and development helps in planning and managing the irrigation timing in regions prone to drought or dependent on rainfall. Therefore, we hypothesized that different soil moisture levels during the early vegetative stage would impact corn physiology, growth, and development. The objectives of the study were (i) to quantify the effects of different levels of soil moisture content on corn physiology and early-season growth and development and (ii) to develop functional relationships between soil moisture content and root and shoot traits that best explain the plant responses to different soil moisture regimes.

## 2. Results

**Figure 1:**
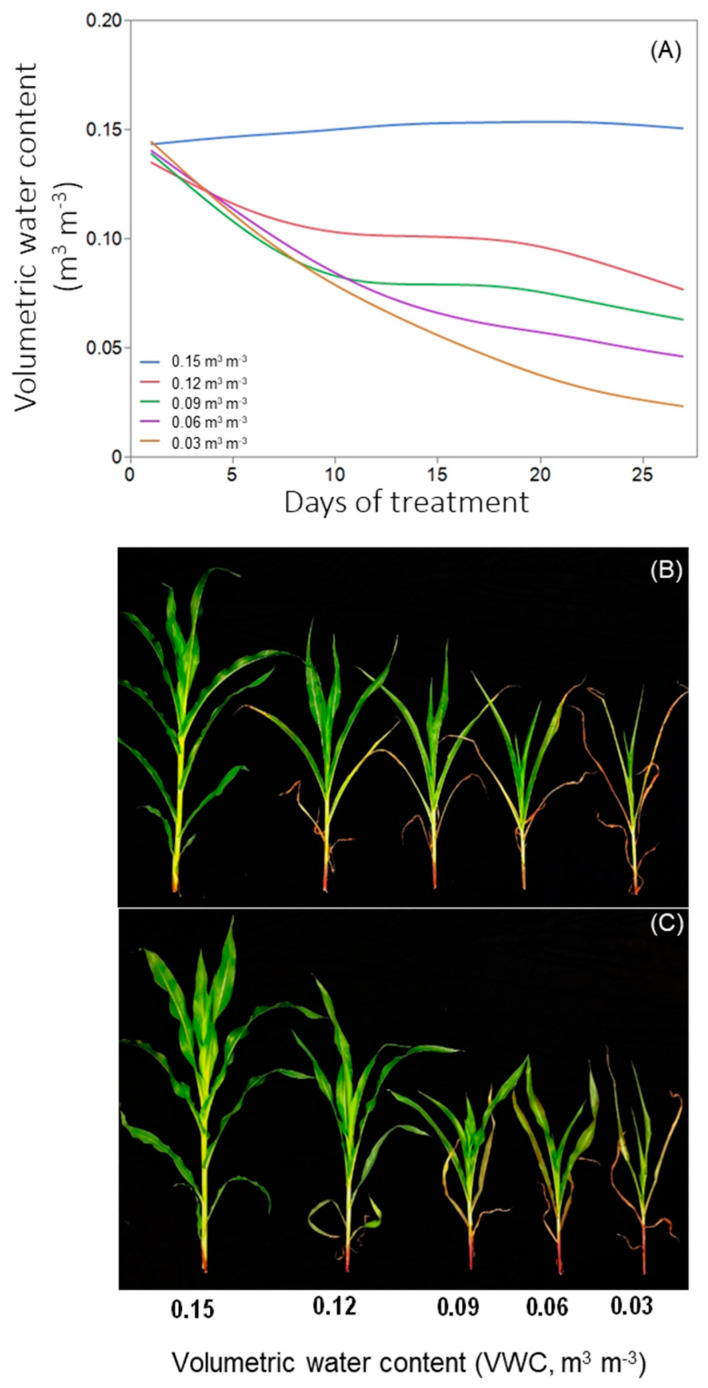
Temporal trends in volumetric soil moisture content across treatments during the corn experimental period (**A**). Pictorial representation of soil moisture stress effects on ‘A6659VT2RIB’ (**B**) and ‘P1316YHR’ (**C**) illustrating plant vigor. The pictures were taken at the final harvest, 28 days after soil moisture stress treatments (38 days after planting).

Soil moisture treatments significantly affected the physiology, pigments, and biomass-related traits of corn hybrids (‘A6659VT2RIB’ and ‘P1316YHR’) in experiments 1 and 2. With increased stress intensity (Figure 1A), plant growth declined in both corn hybrids (Figure 1B,C).

### 2.1. Physiological Parameters

**Figure 2:**
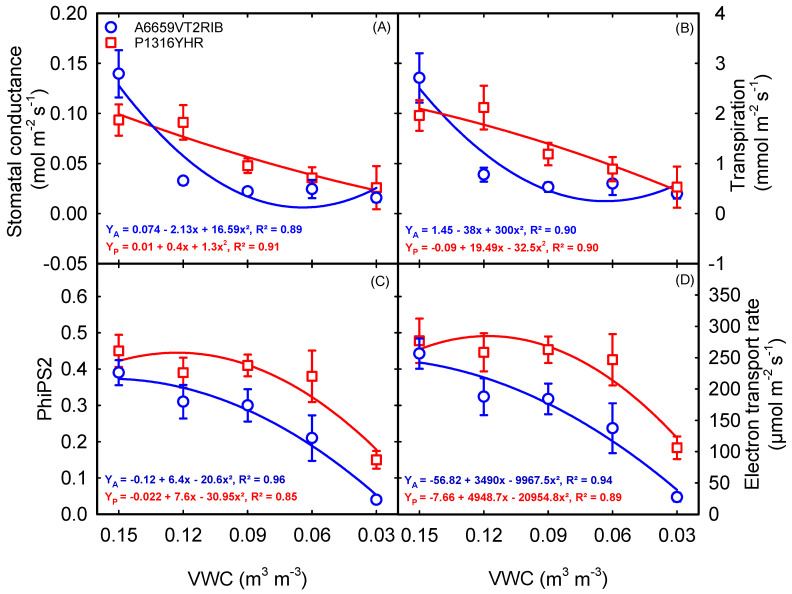
Relationship between volumetric water content (VWC) and stomatal conductance (**A**), transpiration (**B**), the quantum efficiency of photosystem II (PhiPS2) (**C**), and electron transport rate (**D**). Each data point represents mean ± SE.

**Figure 3:**
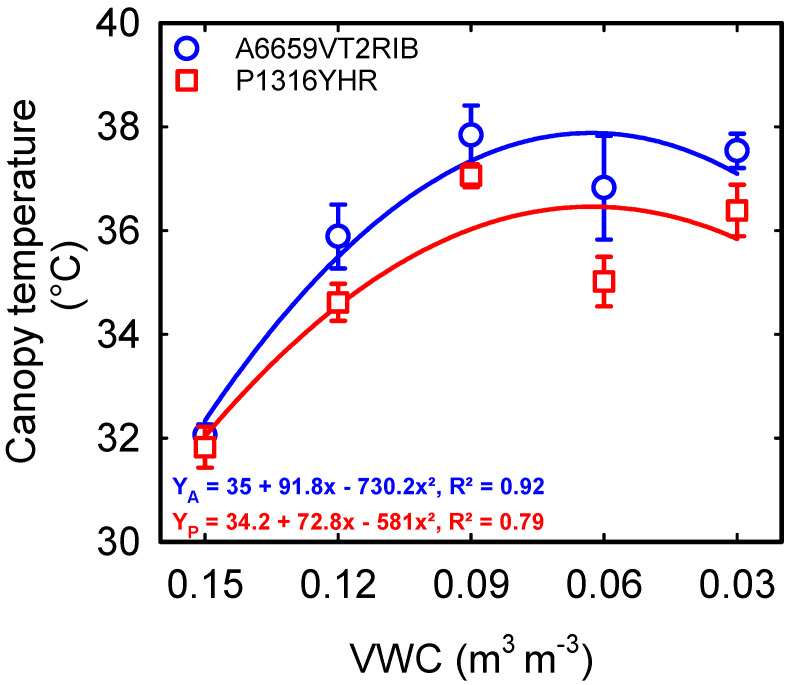
Relationship between volumetric water content (VWC) and canopy temperature. Each data point represents mean ± SE.

A significant interaction between soil moisture content and hybrid (*p* < 0.05) was observed for stomatal conductance and transpiration during the early vegetative growth (Table 1). At the same time, the electron transport rate (ETR) and quantum efficiency of photosystem II (PhiPS2) had a significant treatment (*p* < 0.001) and hybrid effect (*p* < 0.001) (Table 1). A decrease in the stomatal conductance or transpiration was recorded in both hybrids, with declining soil moisture content (Figure 2A,B). However, corn hybrid ‘A6659VT2RIB’ showed an exponential decrease in stomatal conductance and transpiration from control (0.15 m^3^ m^−3^ VWC) to 0.12 m^3^ m^−3^ VWC, which was not the case for ‘P1316YHR’. Under optimum soil moisture conditions (0.15 m^3^ m^−3^ VWC), hybrid ‘A6659VT2RIB’ recorded higher stomatal conductance and transpiration than ‘P1316YHR’ (Figure 2A,B). However, under extreme soil moisture deficit (0.03 m^3^ m^−3^ VWC), the decrease in stomatal conductance and transpiration were seven-fold for ‘A6659VT2RIB’ and four-fold for ‘P1316YHR’ compared to the control. PhiPS2 and ETR differed across the soil moisture treatments and hybrids. PhiPS2 was negatively impacted, with a 78% reduction under 0.03 m^3^ m^−3^ VWC to control (Figure 2C). On average, ETR declined by 48% across stress treatments in ‘A6659VT2RIB’ (R^2^ = 0.94) and by 21% in ‘P1316YHR’ (R^2^ = 0.89) (Figure 2D). The canopy temperature was raised with increased severity of soil moisture deficit in both the corn hybrids. Averaged across hybrids, the non-stressed plants (0.15 m^3^ m^−3^ VWC) had a 5 °C cooler canopy than the plants under severe soil moisture deficit (0.03 m^3^ m^−3^ VWC) (Figure 3).

### 2.2. Pigments

**Figure 4:**
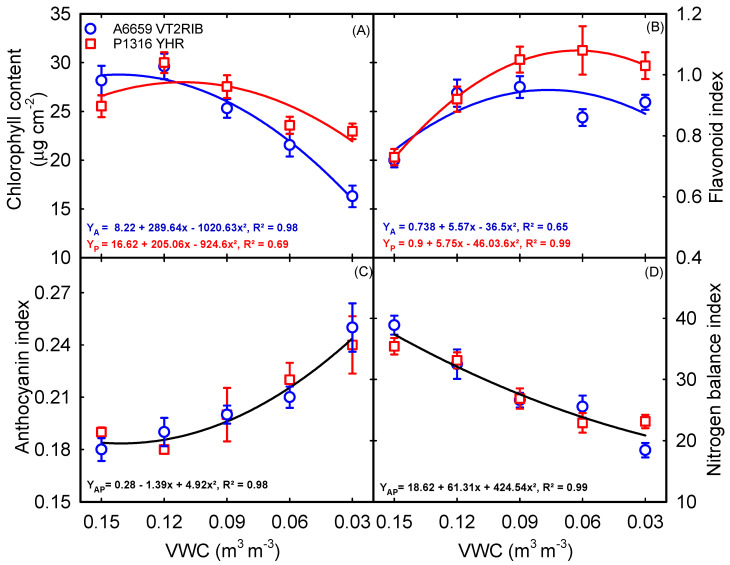
Relationship between volumetric water content (VWC) and chlorophyll content (**A**), flavonoid index (**B**), anthocyanin index (**C**), and nitrogen balance index (**D**). Each data point represents mean ± SE.

Soil moisture treatments significantly (*p* < 0.001) affected the chlorophyll content, flavonoid index, anthocyanin index, and nitrogen balance index (NBI) (Table 1). Further, the interaction between hybrid and soil moisture content was significant for chlorophyll content (*p* < 0.01) and flavonoid index (*p* < 0.05) (Table 1). A decrease in chlorophyll content in both hybrids was associated with increased soil moisture deficit (Figure 4A). However, at 0.12 m^3^ m^−3^ VWC, ‘A6659VT2RIB’ (5%) and ‘P1316YHR’ (18%) had relatively higher chlorophyll content than with the 0.15 m^3^ m^−3^ VWC (Figure 4A). Unlike chlorophyll content, higher flavonoid and anthocyanin were observed across both hybrids under the severe soil moisture deficit conditions (0.03 m^3^ m^−3^ VWC) (Figure 4B,C). The hybrid ‘P1316YHR’ accumulated a higher flavonoid index under soil moisture deficit than ‘A6659VT2RIB’. There was a decrease in the NBI (ratio of chlorophyll content to flavonoid index) across the hybrids with increased stress severity due to decreased chlorophyll content and increased flavonoid index (Figure 4D). A decreasing trend in the NBI of both hybrids was also associated with an increase in the anthocyanin index.

### 2.3. Plant Height, Number of Leaves, and Whole Plant Leaf Area

**Figure 5:**
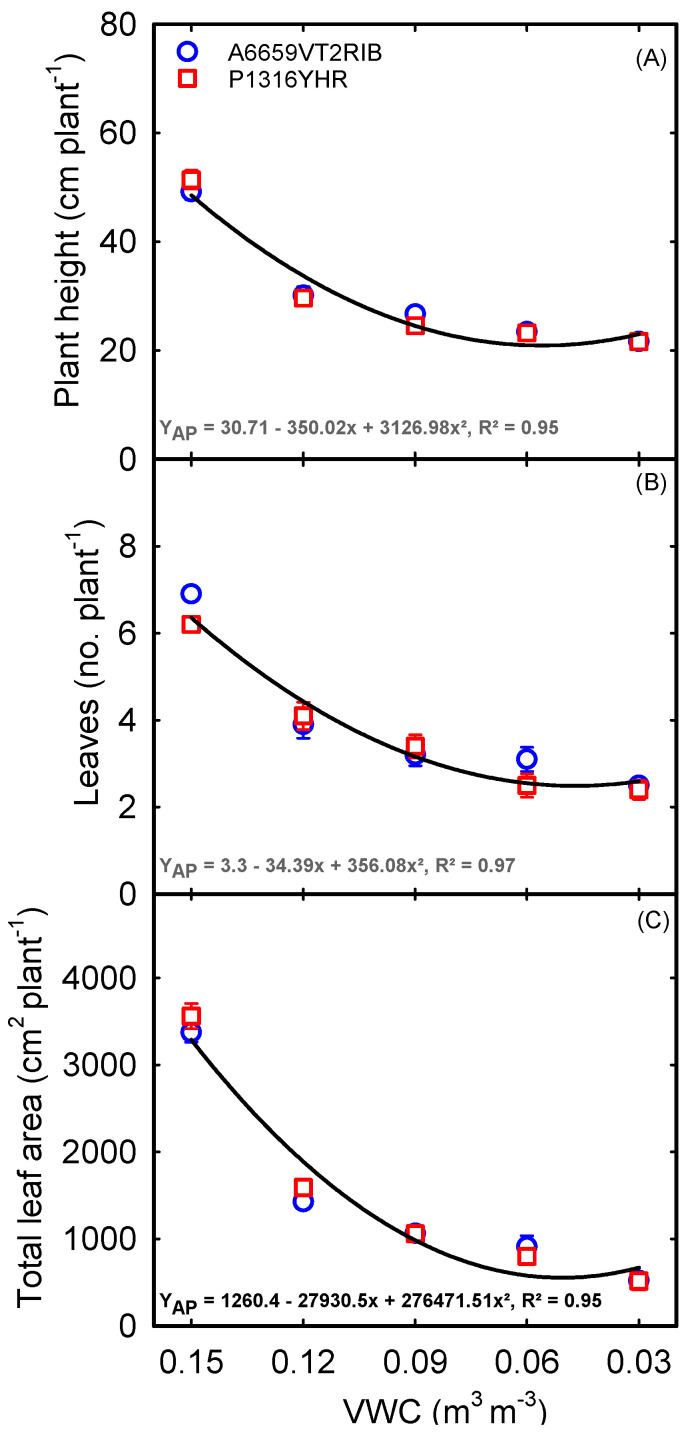
Relationship between volumetric water content (VWC) and plant height (**A**), leaf number (**B**), and total leaf area (**C**) in corn. Each data point represents mean ± SE.

Plant height, number of leaves, and whole plant leaf area were significantly affected (*p* < 0.001) by treatment (Table 1). Decreasing soil moisture content reduced plant height (Figure 5A), leaf number (Figure 5B), and total leaf area (Figure 5C). The average number of fully opened leaves were seven per plant across hybrids under control conditions (0.15 m^3^ m^−3^ VWC). In contrast, there were three fully opened leaves per plant under severe soil moisture deficit (0.03 m^3^ m^−3^ VWC) (Figure 5B). Averaged across hybrids, a maximum total leaf area of 3468 cm^2^ was observed under non-stress conditions. The total leaf area was reduced by 57% under 0.12 m^3^ m^−3^ VWC while it was reduced by 85% to 520 cm^2^ under 0.03 m^3^ m^−3^ VWC (Figure 5C). Quadratic decline in plant height (R^2^ = 0.95), leaf number (R^2^ = 0.97), and total leaf area (R^2^ = 0.95) were observed across hybrids (Figure 5).

### 2.4. Biomass Parameters

**Figure 6:**
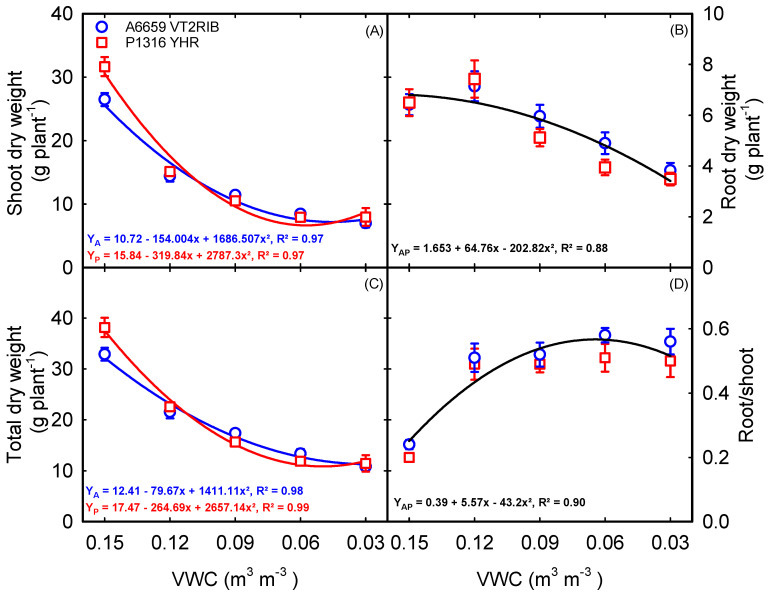
Relationship between volumetric water content (VWC) and shoot dry weight (**A**), root dry weight (**B**), total dry weight (**C**), and root-to-shoot ratio (**D**). Each data point represents mean ± SE.

Soil moisture deficit significantly affected the accumulation of total dry weight (shoot and root dry weight) in both hybrids (Table 1). Quadratic functions best described biomass components with declining soil moisture content in both hybrids (Figure 6). A reduction in shoot dry weight was associated with increased soil moisture deficit (Figure 6A). Corn hybrids grown under less than 0.15 m^3^ m^−3^ VWC produced a low amount of above-ground shoot dry weight (leaf and stem weight) (Figure 6A). Mild soil moisture deficit (0.12 m^3^ m^−3^ VWC) favored the root growth, resulting in an increase in dry weight of 11% in ‘A6659VT2RIB’ and 14% in ‘P1316YHR’ compared to non-stress conditions (0.15 m^3^ m^−3^ VWC) (Figure 6B). The reduction in irrigation supply resulted in a marked loss of overall plant dry matter accumulation (biomass) (Figure 6C). On average, across hybrids, the root weight reduction was at a minimum of 0.09 m^3^ m^−3^ VWC (15%), while the maximum reduction was recorded at 0.03 m^3^ m^−3^ VWC (43%). Total dry matter accumulation (shoot and root dry weight) ranged between 12 g and 36 g from low to optimum soil moisture content (Figure 6C). The root-to-shoot ratio increased quadratically due to a decline in soil moisture content. Under non-stress conditions, the lowest root-to-shoot ratio was observed for both hybrids (Figure 6D). An increased soil moisture deficit ranging from 0.15 to 0.03 m^3^ m^−3^ VWC increased the root-to-shoot ratio from 0.22 to 0.52 (R^2^ = 0.90) (Figure 6D).

### 2.5. Stress Tolerant Index

**Figure 7:**
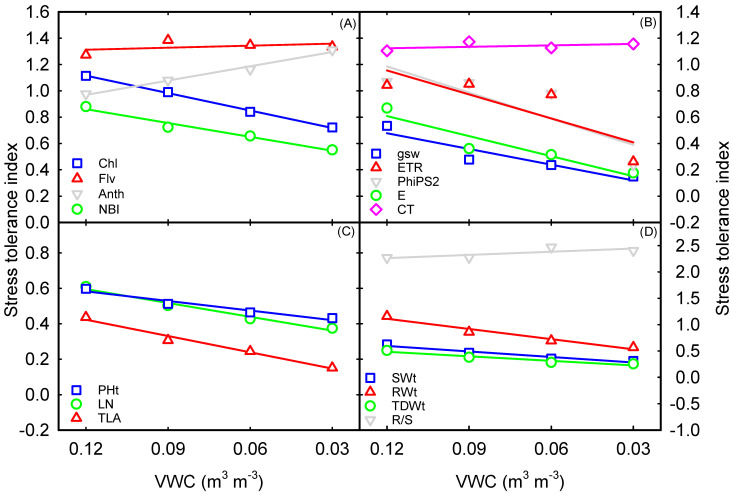
Relationship between volumetric water content (VWC) and shoot dry weight (**A**), root dry weight (**B**), total dry weight (**C**), and root-to-shoot ratio (**D**) measured 28 days after soil moisture treatments. Each data point represents mean ± SE of both hybrids. VWC−Volumetric water content, Chl−chlorophyll content, Flv−flavonoid index, Anth−anthocyanin index, NBI−nitrogen balance index, gsw−stomatal conductance, ETR−electron transport rate, PhiPS2−quantum efficiency of PSII, E−transpiration, CT−canopy temperature, PHt−plant height, TLA−total leaf area, LN−leaf number, SWt−shoot dry weight, RWt−root dry weight, TDWt−total dry weight, and R/S−root-to-shoot ratio.

The stress tolerance index (STI) was calculated and averaged for all the measured parameters across hybrids to understand the trait plasticity. Chlorophyll content and NBI had negative relationships with soil moisture treatments. On the other hand, the anthocyanin index displayed a positive relationship with soil moisture deficit, and the flavonoid index showed no change (Figure 7A). A linear decline occurred for stomatal conductance and transpiration with increased soil moisture deficit (Figure 7B). In response to a decrease in soil moisture content, the STI of all shoot parameters were linearly decreased (Figure 7C). Under various soil moisture deficit conditions, the STI of root dry weight (1.16–0.57) was relatively higher than that of shoot dry weight (0.51–0.25), where the root-to-shoot ratio increased with declining soil moisture content (Figure 7D). Physiology, above-ground growth, and developmental traits were the most sensitive or vulnerable traits among the studied parameters.

## 3. Discussion

The optimum growing conditions during the vegetative stage of row crops favor the expansion of leaf area and, thus, the accumulation of plant dry weight. Drought stress at the vegetative stage delays leaf formation and reduces leaf area, subsequently reducing plant development and crop establishment. Therefore, rainfed-grown corn yields are often lower compared to irrigated corn. Previous studies indicated the relevance of drought stress tolerance at the early seedling stage [32,33,34]. However, no systematic studies have investigated the functional relationships between plant phenotype and soil moisture regimes in corn. The uncertainty in creating and maintaining gradient soil moisture conditions in field conditions has been a significant bottleneck when addressing drought tolerance in corn. To the best of our knowledge, this is the first study demonstrating the morpho-physiological traits responses of corn hybrids under a wide range of soil moisture content levels at the early vegetative stage.

Plants modify their morphology and physiological functions in response to stress. The ability of plants to adapt to soil moisture stress depends on the intensity, duration, and growth stage [35]. Among many traits enumerated in the literature, resource capture and use efficiency traits have great relevance at the vegetative stage. Therefore, in this investigation, we utilized two hybrids to examine the variations in response to the soil moisture content of above-ground and below-ground traits. In this study, reduced soil moisture content for 28 consecutive days decreased stomatal conductance and transpiration by 65% and 59% (Figure 2A,B). These modifications increased the canopy temperature by 5 °C under the lowest soil moisture content (Figure 3). Overall, ‘P1316YHR’ had better stomatal conductance and transpiration than ‘A6659VT2RIB’ across treatments (Figure 2A,B). Similar responses to drought were observed in soybean, where withholding water for a week reduced stomatal conductance by 83% [36], early-season drought reduced stomatal conductance by 74% in sweet potato [37], and in sorghum by up to 72% [38]. These stomatal limitations reduce gas exchange, Rubisco activity, and net photosynthesis under drought conditions [11,39,40].

Leaf photosynthetic pigments are essential for light harvesting in the photosynthetic processes and energy production. Modified physiological traits and photosynthetic pigments under drought conditions are associated with reduced leaf area (leaf number and expansion) and, in turn, plant dry matter accumulation during early vegetative growth. In this study, on average, chlorophyll content was increased by 11% under mild soil moisture deficit, whereas it declined significantly under severe stress conditions in ‘A6659VT2RIB’ (Figure 4A). The decline in chlorophyll content might be due to stress-induced chlorophyll degradation, photo-oxidation, and chlorophyll synthesis deficiency [41]. Further, a reduction in chlorophyll content also affects the light-harvesting capacity of chloroplast by impacting PhiPS2, and ETR, under severe soil moisture deficit conditions [42]. In this study, severe soil moisture deficit (0.03 m^3^ m^−3^ VWC) reduced PhiPS2 and ETR by 78% and 76%, respectively (Figure 2C,D). Subsequently, an increase in the anthocyanin and flavonoid index can control oxidative damage in plants caused by reactive oxygen species (ROS) under stress [43,44]. For instance, in this study, the flavonoid index increased by 39% under soil moisture deficit in ‘P1316YHR’, indicating that flavonoids are drought stress-responsive (a positive sign that can be used as a proxy for drought stress tolerance). On the other hand, both corn hybrids reduced biomass and leaf area, which is a primary response to moisture stress during early growing seasons.

Rapid leaf expansion is essential for a balanced CO_2_ and H_2_O flux, maximizing solar radiation interception and biomass accumulation [45]. However, drought significantly reduces leaf growth and development by limiting cell division or development and expansion. In the present study, leaf parameters revealed a significant decline in terms of total leaf number (Figure 5B) and total plant leaf area (70%) (Figure 5C) in both hybrids under drought stress, indicating the limitation associated with the photosynthetic capacity and source assimilation (biomass). Reduced leaf area is recognized as a drought response during the early season in crops. This slower leaf growth not only delays phenological development but also plays a crucial role in plant survival under limited soil moisture conditions by minimizing the water loss [37,46]. Research has shown that under moderate drought conditions, a slower rate of leaf development can lead to improved root growth, which in turn enhances water uptake. Nevertheless, it is crucial to note that as the severity of drought intensifies, the rate of leaf senescence accelerates, resulting in a detrimental effect on biomass production [12,47]. Both corn hybrids displayed a similar response for leaf traits across soil moisture gradients, with notable reductions observed in leaf area (0.12–0.03 m^3^ m^−3^ VWC) and leaf numbers (0.03 m^3^ m^−3^ VWC). These leaf responses suggest the importance of systematic analysis in identifying differential plant responses depending on severity. The study also highlights the significance of leaf development in maintaining phenotypic plasticity under drought conditions for efficient water use and biomass accumulation.

Vigorous plant growth is critical in achieving higher yields. The overall biomass potential of corn is determined by combining the shoot and root dry weights. A decrease in leaf area, leaf number, and final shoot dry weight resulted in a significant decrease in above-ground biomass. As expected, both corn hybrids exhibited higher biomass under control than under stress treatments (Figure 6). Early-season root vigor increases the water and nutrient uptake, which helps the plant to survive in a stressful environment [48]. Under water deficit conditions, roots play a critical role in above-ground biomass production. Increased soil moisture deficit during the vegetative stage significantly affected root biomass. An increase of 13% in root dry weight compared with control was recorded under mild drought stress (0.12 m^3^ m^−3^ VWC), and as the severity of stress increased, the root dry weight was also reduced by 30%. (Figure 6B). A greater reduction in shoot weight than root weight under drought conditions was related to an increased root-to-shoot ratio [47]. Root dry weight shows a higher stress tolerance index than shoot dry weight, indicating that shoot traits are highly responsive to drought stress. Regardless of soil moisture treatments, ‘P1316HR’ demonstrated higher stomatal conductance and lower canopy temperature than ‘A6659VT2RIB’.

On the other hand, ‘A6659VT2RIB’ showed a relatively higher root weight than ‘P1316YHR’ under extremely low soil moisture content. Our findings suggest that slow above-ground growth with a vigorous root system under stress conditions would improve water uptake and use efficiency during early vegetative growth in corn. An extended study of systemic quantification of biochemical and metabolic responses is necessary to understand the tradeoff or resource allocation between root and shoot under drought stress.

## 4. Materials and Methods

### 4.1. Plant Materials and Growth Conditions

Two independent outdoor experiments were conducted using the pot-culture facility at the R. R. Foil Plant Science Research Center, Mississippi State University, Mississippi State, MS, USA (33° 46′ N, 88° 78′ W) following the institutional guidelines. Two commercial corn hybrids, Agrigold ‘A6659VT2RIB’ (Agrigold Inc., St. Francisville, IL, USA) and Pioneer ‘P1316YHR’ (Pioneer Hi-Bred, Johnston, IA, USA), treated with fungicide were used in this study. The pots (15 cm diameter and 45 cm height) were filled with fine sand and ground farm soil in a 3:1 ratio (sandy loam soil). Two independent experiments were performed with uniform growing degree days (GDD). Each pot was sown with four seeds, and upon emergence, they were thinned to one plant per pot. A total of 100 plants (two experiments × two hybrids × five treatments × five replicates) were used in this study. The pots were arranged in a twin-row orientation on an elevated wooden plank placed on the concrete platform. Mini rain-out shelters, with transparent plastic sheets allowing photosynthetic active radiation (PAR), were set over pots during treatment to avoid precipitation interference [49]. The microclimatic conditions were monitored by installing HOBO data loggers (Onset computer corporation, Bourne, MA, USA). Each pot was set up with drippers with a discharge rate of 50 mL min^−1^ and irrigated with full-strength Hoagland’s nutrient solution maintaining optimal soil moisture conditions (0.15 m^3^ m^−3^ VWC) until the initiation of stress [50].

### 4.2. Soil Moisture Treatments

Plants were grown under optimum soil moisture conditions until they reached the V2 stage. Upon reaching the V2 stage, treatments were applied involving five different levels of soil moisture content with 0.15 m^3^ m^−3^ VWC representing 100% irrigation (control) and 0.12, 0.09, 0.06, and 0.03 m^3^ m^−3^ VWC as 80, 60, 40, and 20% irrigation of control representing soil moisture deficit treatments, respectively (Figure 1A). Soil moisture regimes were used to mimic the drought classification system provided by the United States Drought Monitor Index [51] along with control during early vegetative growth. Soil moisture content was monitored and maintained at desired levels using a real-time soil moisture sensor inserted in the pots (ECH2O, EM5B Soil Moisture, Decagon Devices, Inc., Pullman, WA, USA) and theta probe ML2x soil moisture sensors (Delta-T Devices, Cambridge, UK). Based on the measured VWC, the required amount of water with the nutrient solution was applied for each treatment for 28 days [37].

### 4.3. Data Collection

All non-destructive measurements, such as pigments and physiological data, were collected twice (at 27 and 28 days after stress) per experiment on a sunny day between 10:00 and 13:00 h. Data for determining both physiological and morphological traits discussed were collected from all the replicates within each treatment for both hybrids. All the non-destructive data were collected from the uppermost fully opened leaf of each plant. In both experiments, biomass parameters were collected 28 days after the stress period or 38 days after sowing.

#### 4.3.1. Physiological Parameters

Stomatal conductance (mol m^−2^ s^−1^), transpiration (mmol m^−2^ s^−1^), PhiPS2, and ETR (μmol m^−2^ s^−1^) were recorded using a portable LI-600 porometer integrated with a fluorometer (LI-COR Biosciences, Lincoln, NE, USA). Leaf pigments such as chlorophyll content (μg cm^−2^), flavonoid index, anthocyanin index, and nitrogen balance index (NBI) were recorded using a handheld Dualex optical leaf clip meter (Force A DX16641, Paris, France). Canopy temperature (°C) was measured using an infrared radiometer (Apogee Instruments, Logan, UT, USA).

#### 4.3.2. Growth and Developmental Parameters

Plant height (cm plant^−1^) was measured with a meter scale from the base of the stem to the uppermost fully developed collar leaf. Fully expanded leaves (no. plant^−1^) for each plant were counted manually. Leaves were separated from the stem. The plant’s total leaf area (cm^2^ plant^−1^) was measured using an LI-3100 leaf area meter (LiCOR Biosciences, Lincoln, NE, USA). Roots were washed thoroughly using a wire mesh sieve with a slow water stream. Plant component (leaf, stem, and root) samples were bagged separately and oven-dried at 60 °C for five days. Shoot dry weight (g plant^−1^) was calculated by summing up the stem and leaf dry weights. Total dry matter (g plant^−1^) was calculated by adding shoots and roots together (g plant^−1^). The root-to-shoot ratio was calculated by dividing root dry weight to shoot dry weight.

### 4.4. Statistical Analysis

Two independent experiments were conducted in a completely randomized design with two hybrids, five treatments, and five replications for each treatment. In both experiments, no significant variation was observed in overall growth and biomass accumulation within hybrids at the treatment level. They were grown under uniform growing degree days, so the data from the two experiments were pooled at the replication level (Table S1). The data were analyzed using two-way ANOVA in R studio version 4.2. [52] to evaluate the significant effects of treatments on hybrids. When the F-probability value was significant, the mean values of plant responses were compared using Fisher’s LSD test with *p* < 0.05. Regression analysis was performed using Sigmaplot version 14.5 (Systat Software Inc., San Jose, CA, USA) to check both hybrids’ quadratic treatment responses. Graphs were generated using Sigmaplot version 14.5. The stress tolerance index [53] was used to calculate each parameter at different soil moisture levels.

STI = (Y_P_) × (Y_S_)/(Ŷ_P_)^2^ (1)

where

Y_P_ is the response of an individual hybrid under control conditions for a given parameter.

Y_S_ is the response of individual hybrid response under stress conditions for a given parameter.

Ŷ_P_ is the mean response of hybrids under control conditions for a given parameter.

STI results of collected parameters across hybrids were averaged and further grouped into pigment, physiological, leaf, and biomass categories to understand the relative effect of reduced soil moisture content on plant traits.

## 5. Conclusions

The current study investigated the physiology, morphology, and biomass responses to various soil moisture levels during the early-season growth (V2 to V7) stage in corn. The results suggest that corn plants employ varying adaptive strategies to cope with drought conditions, with a primary focus center around energy conservation. There was a notable reduction in physiological and growth-related traits, these responses often accompanied by concurrent improvements in resource use efficiency, particularly during the early vegetative stage. Non-linear trait responses were observed, regardless of the linear decline in soil moisture content, depending on the hybrid. The stress tolerance index revealed that physiological and leaf parameters exhibited greater sensitivity to soil moisture stress compared to other traits. In addition, the study indicated that the allocation of biomass between above-ground and below-ground traits dynamically changed with stress intensity. The functional relationships developed can be used to predict plant performance and improve agronomic practices under stressful conditions. Furthermore, the phenotypic data generated from this study can assist in prioritizing traits associated with early-season drought tolerance in corn. By prioritizing traits associated with drought tolerance, breeders can develop resilient corn that can withstand soil moisture stress during critical growth stages.

## Figures and Tables

**Table 1:** Summary of the analysis of variance of soil moisture stress effects on physiological, growth, and developmental traits in corn.

Source	gsw	E	ETR	PhiPS2	CT	Chl	Flv	Anth	NBI	PHt	TLA	LN	SWt	RWt	TDWt	R/S
Hybrid (H)	ns	ns	***	***	**	*	**	ns	ns	ns	ns	ns	ns	ns	ns	ns
Treatment (T)	***	***	***	***	***	***	***	***	***	***	***	***	***	***	***	***
H × T	*	*	ns	ns	ns	**	*	ns	ns	ns	ns	ns	**	ns	*	ns

Stomatal conductance (gsw, mol m^−2^ s^−1^), transpiration (E, mmol m^−2^ s^−1^), electron transport rate (ETR, μmol m^−2^ s^−1^), the quantum efficiency of PSII (PhiPS2), canopy temperature (CT, °C), chlorophyll content (Chl, μg cm^−2^), flavonoid index (Flv), anthocyanin index (Anth), nitrogen balance index (NBI), plant height (PHt, cm), total leaf area (TLA, cm^2^ plant^−1^), number of leaves (LN, no. plant^−1^), shoot dry weight (SWt, g plant^−1^), root dry weight (RWt, g plant^−1^), total dry weight (TDWt, g plant^−1^), and root-to-shoot ratio (R/S). ***, **, *, and ns indicate significance at *p* < 0.001, *p* < 0.01, *p* < 0.05 and non-significant effect, respectively.

## Data Availability

All the materials and data generated in this study are included in this article, or the corresponding author can provide any additional data required upon request.

## Supplementary Materials

The following supporting information can be downloaded at: https://www.mdpi.com/article/10.3390/plants12132471/s1, Table S1: Summary of non-significant experiment × treatment interaction across biomass traits. Values indicate the coefficient of correlation (R^2^) of quadratic regression between soil moisture content and trait interest.
