# Supplementary material for: Developing Functional Relationships between Soil Moisture Content and Corn Early-Season Physiology, Growth, and Development
Source: Plants (Basel). 2023 Jun 28;12(13):2471. doi: 10.3390/plants12132471 (PMC10346487; doi:10.3390/plants12132471)
Supplement: Supplementary file 1 [file plants-12-02471-s001.zip › plants-2468884-supplementary.pdf]

Table S1 Summary of non-significant experiment × treatment interaction across biomass traits. Values indicate the coefficient of correlation (R^2) of quadratic regression between soil moisture content and biomass-related traits.

Trait	Experiment × Treatment	A6659VT2RIB (R^2)		P1316YHR (R^2)	
		Experiment 1	Experiment 2	Experiment 1	Experiment 2
Total leaf area (cm^2 $plant^{-1}$)	ns	0.9	0.96	0.97	0.95
Shoot dry weight (g $plant^{-1}$)	ns	0.95	0.98	0.97	0.98
Root dry weight (g $plant^{-1}$)	ns	0.96	0.91	0.95	0.71
Total dry weight (g $plant^{-1}$)	ns	0.97	0.99	0.99	0.99
